# The chronotype conjecture in the association between dietary carbohydrate intake and high-sensitivity C-reactive protein (hs-CRP): a cross-sectional study from NHANES 2015 data

**DOI:** 10.5935/1984-0063.20200047

**Published:** 2021

**Authors:** Raissa Victorine Ngo-Nkondjock, Zhang Yuntao, Humara Adnan, Sheikh Muhammad Adnan, Thérèse Martin Wabo Cheteu, Ying Li

**Affiliations:** 1 Harbin Medical University, Department of Nutrition and Food Hygiene, School of Public Health - Harbin - Heilongjiang - China.; 2 Harbin Medical University, Department of Epidemiology and Biostatistics, School of Public Health - Harbin - Heilongjiang - China.; 3 COMSATS University, Department of management sciences - Islamabad - Pakistan.

**Keywords:** Carbohydrates Intake, Cardiovascular Health, Dietary Carbohydrates, Cardiovascular Diseases, Ethnicity and Health

## Abstract

Substantial evidence suggests that the timing of macronutrient intake affects cardiovascular health. The present study aims to assess the association between the dietary carbohydrate intake (DCI) and the high-sensitivity C-reactive protein (hs-CRP) combined with the implication of the chronotype. Thus, we explored the most recently released National Health and Nutrition Examination Survey (NHANES) data. We analysed data from 5,616 participants of the NHANES in 2015. We selected participants with available data for the DCI, sleep and wake-up time, and the hs-CRP. Chronotypes were categorized according to the sleep times. Binary logistic regression analysis was performed to predict participants with low or high levels of hs-CRP based on the DCI and chronotypes. Moderation analysis was used to investigate the effect of the chronotypes on the DCI-hs-CRP’s association. A higher DCI was significantly associated with the higher hs-CRP levels (odds ratio (OR) = 1.36, 95% confidence interval (CI) = [0.9-1.8]). Moderate evening (ME) chronotypes had higher risk for high hs-CRP level (OR = 1.15, 95% CI = [1.22-1.23]) compared to the intermediate and the morning chronotypes. The chronotype significantly moderated the hs-CRP given the DCI (moderation coefficient, α2=0.05, 95% CI = [0.01-0.08]). The chronotype diminished the hs-CRP predicted by the DCI. The findings of the study underscore the significance of assessing the protective effect of individuals’ chronotype concerning cardiovascular health.

## INTRODUCTION

Nutrition imported from one culture to another, and increased and advanced social networks, as well as the management of leisure time within families^1,2^ result in changes in people’s aspects of lifestyle. These changes harm communities, such that an individual’s behavioural pattern may change and become non-beneficial for his overall health and well-being^3^. One significant factor that influences behaviour patterns is chronotype, defined as the circadian typology of an individual^4^. It is a behavioural manifestation of an individual’s internal circadian clock system, which can be assessed with the use of multiple methodologies that classify individuals’ quality of sleep. From 2006 to 2016, the frequency of deaths due to cardiovascular disease decreased by 18.6% in the US, but still, multiple factors hamper the efforts toward achieving the goal of eradicating them^5^. Among such factors, there are self-regulated systems like chronotype and chrononutrition, any change in which the cycle of body cells may be altered, causing cardiovascular disease over the time^6-8^.

Many research studies on the association of carbohydrate consumption with the risk of developing heart disease have shown that the regular intake of carbohydrate increases the risk of cardiovascular disease^9-11^. Other studies have also indicated that individuals’ waking and sleeping times may impact on their pattern of carbohydrate consumption^12-15^. Furthermore, a secondary analysis of clinical trial data pointed out the controversy over the quality of people’s sleep and the risk of cardiovascular disease. The results of this study showed an association between long sleep duration and the risk of developing a stroke. The concerned group is made up of patients living with cardiovascular disease and presenting sleep-apnea disorders at the same time^16^.

On the other hand, studies that focus on cardiovascular health and sleep quality and/or duration often depict an inverse association between the two variables, e.g., poor sleep quality is associated with an increased risk of cardiovascular disease. Another cohort study has revealed an association between short sleep duration (less than 6 hours) and the risk of developing coronary heart disease^17^. In a cross-sectional study of fifty-eight obese men and women, it was observed in the women’s group that, sleep duration and food composition were not associated. The results of the study also suggest an inverse association between protein intake (r=-0.43; *p*=0.02), monounsaturated fatty acids (r=-0.40; *p*=0.03), cholesterol (r=-0.50; *p*=0.01), and short sleep duration^18^. The present study discusses the impact of the bi-directional sleep-food relationship on cardiovascular health. Indeed, the fact is that having shortened hours of sleep leads to a certain choice of food. Conversely, the choice of food consumed could reflect in the quality of sleep and then cardiovascular health^19^.

The reciprocity evoked in the food-sleep relationship raises the question of sleep mediation in the association between diet and cardiovascular health. Thus, the present study aim to investigate the association between the consumption of a specific nutrient with the cardiovascular risk. To carry out our study, we used data from NHANES 2015 with its distinctive touch of ethnic diversity. We considered DCI and hs-CRP records to achieve the following objectives: a) assess how DCI is associated with future risk of developing cardiovascular disease, and b) evaluate the existence of a moderation effect of the participants’ chronotype on the association between the DCI and the hs-CRP.

## MATERIAL AND METHODS

### Source population and sampling

Our population source is the National Health and Nutrition Examination Survey (NHANES) covered by the U.S. Centers for Disease Control and Prevention/National

Center for Health Statistics (CDC/NCHS)^20^. The NHANES proposal is a cross- sectional database representative of the U.S. population. The NHANES conducts bi- annual cycles of data collection and proceeds to public release, following rules and regulations corresponding to the actions related to the use of the data^21^. The 2015-2016 cycle is similar to the 2011-2014 yearly cycles in the variable concerning the races. The NHANES modified this component to clarify the ethnicity of participants who identified themselves as Asians. The 2015-2016 cycle has a full sample of Hispanics (32.1%), White single-race (30.9%), Black single-race (21.5%), Asian single- race (10.3%), and other races including multiracial (5.1%); this cycle is unique in that it includes an hs-CRP variable, an indicator of future risk of cardiovascular disease. We included all participants with available information on dietary carbohydrate intake, wake-up time, and sleep onset time. We ended up with a sample of 5,665 men and women.

### Measurement and assessment of study variables

#### Carbohydrate intake

Carbohydrate intake was measured in grams. Participants responded initially to questionnaires in person and further through phone calls. The questionnaires were both about individual foods and total nutrient intake. We used data collected from the first day and from 24-hour recall on the second day. We excluded all participants with extremely high records of DCI.

#### hs-CRP

We measured the high-sensitivity C-reactive protein (hs-CRP) as a predictor of future cardiovascular disease risk^22^. The hs-CRP was measured with patients in Mobile Examination Centers using the SYNCHRON system, which is based on the methodology of high-sensitivity near-infrared particle immunoassay rates^23^ (CDC- NHANES, 2019). The hs-CRP was measured in mg/l; we used hs-CRP as a categorical variable. Two categories were thus created for the data analysis. And hs-CRP values less than 3mg/l corresponded to the low values group, while the high values group had hs-CRP values greater than or equal to 3mg/l.

#### Chronotype

In identifying participants’ usual bedtime and time of awakening, the following questions were asked: “What time do you usually go to sleep on weekdays or workdays?” and “What time do you usually wake up on weekdays or workdays?”. Questions referred to the times in and out of bed, excluding naps. Responses were reported in the format of 00 to 24 hours and 00 to 59 minutes (HH:MM). Sleep time, if necessary, was rounded up to the next half hour. Individuals were asked to approximate their possible wakefulness during their main sleep periods. Computer-assisted personal interviewing (CAPI) was the system used here to reduce the likelihood of data entry errors. The consistency and completeness of the available information have been scrupulously checked. As part of the data analysis quality control, very extreme sleep times (too low or too high) were confirmed from audio recordings when available^24^.

We categorized the chronotypes utilizing the final scores of the short version of the Morningness-Eveningness Questionnaire (MEQ)^25^. The MEQ, in its simplified version, offers two classification means in order to obtain the scores corresponding to the chronotypes-either the “sleep onset” or the “wake-up offset.” We proceeded to a three-step classification of the chronotypes by selecting cases based on participants sleep and wake-up times. In the first step, we used the participants’ sleep times to classify the chronotypes. Secondly, we referred to the wake-up times for the participants whose sleep times were below and above the ranges that the MEQ scores proposed. Lastly, we considered the extreme early or late values of the sleep onset times as definite morning (DM) or evening chronotypes. We used the participants’ records combined with the MEQ scores, to classify the participants in their chronotypes. Thus, we were able to classify the chronotypes under the five categories proposed in the MEQ scores - “definite morning”, “moderate morning”, “intermediate”, “moderate evening”, and “definite evening (DE)”. The chronotypes were evaluated as a nominal variable.

#### Covariates

The following covariates were used in our analysis: age was categorized as <35, 36-55, or >55 years; sex was categorized as female or male; smoking status was assessed using the question “Do you now smoke?” and categorized as every day, some days, or not at all; the participants self-reported race, we categorized race as “Hispanics”, “non-Hispanic Whites”, “non-Hispanic Blacks”, “non-Hispanic Asians”, and “other including multiracial”; the frequency of alcohol consumption was assessed according to the average number of alcoholic beverages drunk per day for the past 12 months; sleep duration was computed by the sleep onset minus the wake-up time; the systolic blood pressure and diastolic blood pressure of the participants were measured four times and expressed in mmHg, we assessed the systolic and diastolic blood pressures as the mean of four measurements (first measurement + second measurement + third measurement + fourth measurement all divided by 3); the height and weight were assessed through the BMI, we the following formula to calculate participants BMI: as per weight(kg)/height2(cm); the waist circumference was estimated in cm; and the triglyceride level was estimated in mmol/l. To meet an acceptable percentage of analytical data in the covariates^26^, we performed multiple imputations to our dataset before the statistical analysis.

### Statistical analysis

Binary regression was used to estimate the ORs and 95% CIs of the association between dietary carbohydrate intake and the low and high levels of hs-CRP. Carbohydrate intake was categorized by quartile distribution, using the first quartile as reference. In addition to the main effect model, we built both bivariate and multivariate models, respectively model 1 and model 2, for confounders’ control.

Across all chronotypes, binary logistic regression served to predict the hs-CRP levels given the chronotypes. For purposeful analysis, chronotypes were analysed into their categories, and the intermediate chronotype stood for reference. We, therefore, estimated the predictive association, the ORs, and the 95% confidence interval (CI).

We employed the moderation model to analyse the effect of the chronotype on the association between the DCI and the hs-CRP. The moderation model involves the calculation of the total effect of an association and re-estimation of that same total effect when a significant moderator exists; in case the considered moderator effect is significant in the model, there will be a reduction or an increase of the total effect after the moderation coefficient is applied. The α1 coefficient represented the total effect of the dietary carbohydrate without the presence of the moderator. The α2 coefficient corresponded to the chronotype moderation effect on the main effect. The α3 signified the effect of moderation on the main effect. For the moderation model, we analysed quintiles of hs-CRP and low and high levels of DCI.

We applied the strata, cluster and sample weight available according to our data. This preparation for analysis was performed through the complex samples function on SPSS.

Our statistical significance was two-tailed and settled at a *p*-value less or equal to 0.05 provided by the all analysis performed using SPSS 24 (IBM Corp., Armonk, NY, USA) and SPSS PROCESS MACRO version 3.4 by Hayes Andrew that was used to quantify the moderation analysis^27^.

## RESULTS

### Population characteristics

Tables 1 and 2 present the characteristics of our study variables. The DE chronotypes had more participants aged less than 25 years old (27.1%). DCI mean was higher in the DM chronotype group (73g per day). The ME group had higher smoking (55.2%) and alcoholic beverage consumption (5.82 times per day). The DM group presented a higher mean in waist circumference (100.76cm), while systolic blood pressure was more elevated in the definite chronotypes group (126.78). The lowest sleeping duration time was found in the DE group (7.09).

**Table 1:** Continuous variables characteristics.

Frequencies (%)	Definite morning	Moderate morning	Intermediate	Moderate evening	Definite evening	*p*-value
	750	1715	2284^a^	542	325	
Dietary carbohydrates Intake (g)	73.52^a^	73.29	69.07	59.29	48.77	< 0.001
	(70.6)	(70.9)	(73.1)	(64.7)	(54.4)	
Number of Alcoholic Beverage consumed	2.56	3.08	4.31	5.82^a^	2.80	< 0.001
	(7.4)	(13.0)	(36.9)	(50.6)	(4.6)	
Waist circumference (cm)	100.76^a^	99.06	97.98	99.74	98.76	< 0.001
	(15.6)	(16.4)	(17.1)	(18.4)	(18.3)	
Sleep hours	8.56^a^	7.99	7.51	7.12	7.09	< 0.001
	(1.4)	(1.2)	(1.4)	(2.2)	(2.3)	
Systolic blood pressure, mmHg	126.78^a^	123.85	122.64	123.90	123.30	< 0.001
	(18.9)	(17.5)	(17.0)	(18.6)	(17.3)	
Diastolic blood pressure, mmHg	69.15	68.80	68.15	69.07	70.27^a^	< 0.001
	(12.8)	(12.2)	(11.9)	(13.9)	(11.9)	
BMI (%)	29.85^a^	29.16	28.93	23.37	29.55	< 0.001
	(7.1)	(6.8)	(7.1)	(3.5)	(7.5)	
HDL, mmol/l	1.39	1.41a	1.41^a^	1.35	1.35	< 0.001
	(0.5)	(0.4)	(0.4)	(0.4)	(0.4)	

Variables means are given with standard deviation in brackets;

a Highest prevalence group; (*): *p*-values from One Sample T-Test.

**Table 2:** Categorical variables characteristics.

Number of cases	Definite morning	Moderate morning	Intermediate	Moderate evening	Definite evening	*p*- value
	750	1715	2284	542	325	
**Sex (%)**						
Male	49.2	46.5	46.6	52.0	57.5 ^a^	< 0.001
Female	50.8	53.5 ^a^	53.4	48.0	42.5	
**Age in years (%)**						
<25	10.4	16.0	19.2	24.7	27.1 ^a^	< 0.001
26 - 35	14.9	14.4	16.9	17.2	19. 7 ^a^	
36 - 55	34.3 ^a^	34.1	29.7	29.0	31.7	
>56	40.4 ^a^	35.5	34.2	29.2	21.5	
**Race**						
Hispanics	35.9 ^a^	33.1	31.3	26.1	24.6	< 0.001
NHW	32.8	37.4^a^	31.5	24.6	21.5	
NHB	20.7	16.9	19.1	29	34.5^a^	
NHA	8	9.0	13.9	15	15.7^a^	
Other races	2.7	3.6	4.2	5.3^a^	3.7	
**HSCRP**						
Low	64.3	64.5	65.6^a^	61.6	65.5	< 0.001
High	35.7	35.5	34.4	38.4^a^	34.5	
**Smoking status (%)**						
Everyday	34.2	31.5	27.2	52.1	55.2 ^a^	0.001
Someday	9.0	11.7 ^a^	11.7	8.0	6.9	
Not at all	56.8	56.9	61.0^a^	39.9	37.9	

a Highest prevalence group; (*): ^p^-values from Pearson χ^2^.

### Association between DCI and hs-CRP

We performed a binary logistic regression to analyse the ability of the DCI to predict high or low levels of hs-CRP. We analysed the dietary carbohydrate intake in their quartiles, using the first quartile as the reference category. Table 3 shows the results of our analysis. The second, third and fourth quartiles of carbohydrate intake are associated with high hs-CRP levels. Adjustment for age, sex, race, smoking status, alcohol consumption, sleeping duration, BMI, waist circumference, HDL levels, and systolic and diastolic blood pressure showed the same risk association in higher carbohydrate consumers, where OR equals 1.25, 95% CI = [0.9-1.8].

**Table 3:** Association between DCI and low/high levels of hs-CRP.

	1^st^ Quartile	2^nd^ Quartile	3^rd^ Quartile	4^th^ Quartile
**Model 1**				
OR 95% CI	1	1.25 1.1 to 1.5	1.50 1.3 to 1.7	1.45 1.2 to 1.7
**Model 2**				
Exp (B) 95% CI	1	1.19 1.0 to 1.4	1.28 1.1 to 1.5	1.18 0.9 to 1.4
**Model 3**				
Exp (B) 95% CI	1	1.12 0.8 to 1.6	1.36 0.9 to 1.9	1.25 0.9 to 1.8

Model 1: Carbohydrate intake and HSCRP; Model 2: Adjustment with age, sex, and race; Model 3: Adjustment with age, sex, race, smoking status, alcohol consumption, sleep duration, BMI, waist circumference, HDL, systolic and diastolic blood pressure.

### Association between chronotypes and hs-CRP levels

The intermediate chronotypes were the reference category for binary logistic regression analysis. We assessed the association between chronotypes and hs-CRP levels (Table 4). The analysis results revealed a higher risk in the ME chronotypes, OR = 1.18, 95% CI = [0.9-1.4]. After controlling for age, sex, race, smoking status, alcohol consumption, sleeping duration, wake-up time, BMI, waist circumference, HDL levels, and systolic and diastolic blood pressure, the increased risk stayed significant in the ME group, OR = 1.15, 95% CI = [1.22-1.23]. However, there was a similar decreased risk in the DE group OR = 0.82, 95% CI = [1.29-1.30] as in the DM group OR = 0.82, 95% CI = [0.11-0.12].

**Table 4:** Association between chronotypes and low and high hs-CRP levels.

	Definite morning	Moderate morning	Intermediate	Moderate evening	Definite evening
Model 1					
OR 95% CI	1.06 0.9 to 1.3	1.05 0.9 to 1.2	1	1.18 0.9 to 1.4	1.00 0.8 to 1.3
Model 2					
OR 95% CI	0.87 0.92 to 0.93	1.00 1.05 to 1.06	1	1.30 1.32 to 1.33	1.14 0.59 to 0.60
Model 3					
OR 95% CI	0.82 1.29 to 1.30	1.10 1.06 to 1.07	1	1.15 1.22 to 1.23	0.82 0.11 to 0.12

Model 1: Bivariate analysis; Model 2: Adjustment with age, sex, and race; Model 3: Adjustment with age, sex, race, smoking status, alcohol consumption, sleep duration, BMI, waist circumference, HDL, systolic and diastolic blood pressure.

### Chronotypes’ moderation effect on the DCI and the hs-CRP levels

**Figure 1:**
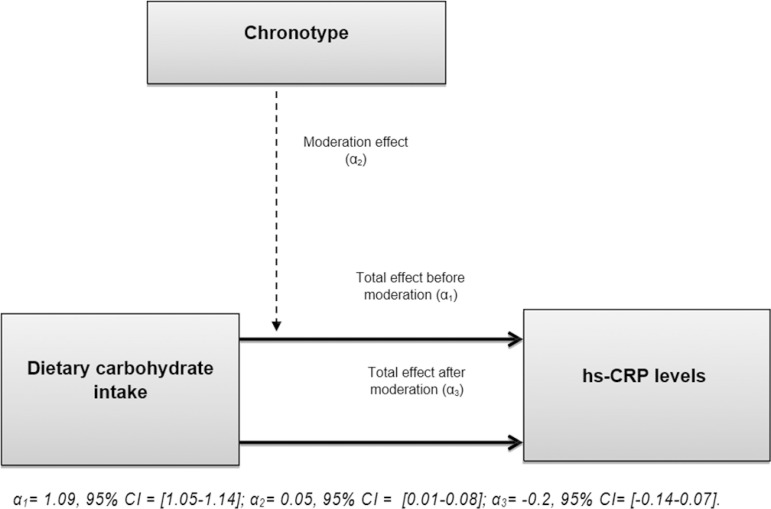
Chronotypes moderation effect

A moderation analysis was performed to analyse the effect of the chronotypes on the association between the DCI and the hs-CRP. For this analysis, we assessed the hs-CRP per their quintiles and the carbohydrates intake per low (less than 250 grams) and high (more than 250 grams) values. The moderation effect coefficient was α2 = 0.05, 95% CI = [0.01-0.08]. The dietary carbohydrate intake and the hs-CRP’s association coefficients before and after moderation were, respectively, α1 = 1.09, 95% CI = [1.05-1.14], and α3 = -0.2, 95% CI = [-0.14-0.07]. The moderation effect is illustrated in Figure 1.

## DISCUSSION

This study analyses the effects of the chronotypes on the association between dietary carbohydrates intake and cardiovascular risk. It was observed that in the group consisting of middle-aged and elderly individuals, there was a higher mean in BMI and waist circumference, which might predict a high cardiovascular risk in the population.

We found in our population that high carbohydrate intake is associated with high hs-CRP levels. The risk remained constant, despite adjustments in sex, age and race, as well as smoking habits, alcohol consumption frequency, blood pressure, BMI, waist circumference, and HDL levels. We included the chronotypes in our analysis, and as a result, our analysis demonstrated that the chronotypes had a moderating effect in the association between the DCI and the cardiovascular risk. Indeed, the chronotype tended to decrease the risk coefficient of the association between DCI and cardiovascular risk.

The strength of the present study relies on the involvement of the moderation effect of chronotypes on the cardiovascular risk in relation with the DCI, the sample size (N=5,665) on which was applied a sample weight to reflect a significant proportion of the entire population of the United States, and the use of the hs-CRP, which is the best indicator of the development of cardiovascular disease in the future to date.

To raise possible limitations of this study, we will mention the single use of the MEQ scoreboard with the sleep unset score ranges for chronotype determination. The MEQ requirements include a set of questions based on sleep onset and wake-up time, in addition to daily base routine points. The entire questionnaire is to be fulfilled before getting the scores that determine one’s chronotype. Handling secondary data means that we did not attain the overall information required by the questionnaire; we therefore used the score ranges from the questionnaire and the sleep time from our dataset to produce the population’s chronotypes. The reliability test of both morningness and eveningness chronotypes is contained in the supplementary material.

Our study is in line with previous studies; moreover, our study adds value to the implication of chronotypes in the association between carbohydrate intake and cardiovascular risk. Furthermore, our study inferred a high level of DCI associated with hs-CRP. Huffman et al.^28^ evaluated the association between dietary carbohydrate intake measured as dietary glycemic load and the plasma hs-CRP in 171 at-risk men and women. In their results, carbohydrate intake was independently related to hs-CRP (r2=0.28; *p*<0.04) in women. Later on, high consumption in dietary carbohydrate intake leading to a significant cardiovascular risk was also demonstrated by Baron et al.^29^, when was found in female individuals an association between late sleeping and carbohydrate consumption, leading to high BMI. Another study has demonstrated the reciprocity of this relationship, i.e., high obesity risk, which tends to be most common in people eating high-energy meals late in the night^30^.

In a context where late chronotypes are associated with cardiovascular-risk behaviours^31,32^, we have surprisingly found in DE a lower risk-association with high hs-CRP compared to earlier chronotypes. Looking at the low frequencies of the DE in our population, we can easily opine the significance of the latter results; analysis with a higher frequency of participants may lead to a different finding. Nevertheless, DE had a higher risk-association with hs-CRP compared to all other chronotypes. In the ME and DE groups, there was respectively higher alcohol consumption and smoking frequencies compared to all other groups. Similarly, Ahola et al.^33^ assessed DCI and cardiometabolic risk factors with type 1 diabetes. A cross-sectional study found that higher carbohydrate intake is associated with several cardiometabolic risk factors such as higher blood pressure, higher waist circumference and lower HDL. However, DM chronotypes presented lower risk factors toward hs-CRP levels, despite a higher waist circumference, systolic blood pressure and BMI. These outcomes highlight a protective effect of the chronotypes on cardiovascular health. The hs-CRP levels were elevated in the ME chronotypes; meanwhile, higher carbohydrate intake was found in the DM group. Our findings are consistent with previous studies in that earlier chronotypes are associated with healthier behaviours and lower cardiovascular risk factors. Among others, Patterson et al.^34^ presented a study in which the morning chronotypes have a tendency to consume more fruits and vegetables, with less screen-based sedentary computer behavior (OR = -0.2, CI = [-0.027 to -0.013]), leading to better heart health.

Chronotype is an inborn factor, and most people belong to the intermediate chronotypes. In our study, the intermediate chronotypes had, on average, fewer associated risk factors related to waist circumference, blood pressure, alcohol consumption, smoking status, and BMI. These findings show that chronotype alone is not a protective or a risk factor, but it is a combination of several factors that ensure optimum health for individuals.

In addition to the association between carbohydrate intake and cardiovascular risk, and the association between carbohydrate intake and chronotypes, a moderating effect of the chronotypes in the association between carbohydrate intake and cardiovascular risk was found in the present study. The presence of the chronotypes diminished the total effect of dietary carbohydrate intake on hs-CRP. Our study is the first to focus on a considerable moderation of the chronotypes on the DCI’s total effect on hs-CRP.

The results of our study suggest that the morning timings modify the relationship between carbohydrate intake and cardiovascular risk. Subsequent observational research, of which controlled clinical trials, will help elucidate better contextual, environmental and behavioural factors that are responsible for the action of chronotypes on the cardiovascular risk induced by high carbohydrate intake. One study mentioned that working on-call, when individuals fill up on all the meals that they skipped during the day leads them to consume fast food, which are high-carbohydrate meals^35^. There are various hypotheses presented together with evidence at this time, but more research on high macronutrient intake is needed to clarify the different hypotheses related to it. Future research will guide decision-making on strategies to change behaviour.

In conclusion, our study showed an association between carbohydrate consumption and cardiovascular risk, plus a moderation effect of the morningness chronotype on the above-cited association. The present study opines the protective effect of the chronotype on cardiovascular health.

## SUPPLEMENTARY MATERIAL

**Appendix (A): Association between carbohydrate intake and chronotypes**

Table A presents the results of the association between carbohydrate intake and chronotypes. Carbohydrate intake was analyzed into their quartiles, with the first quartile as reference. The analysis results show an inverse association between carbohydrate intake and chronotype. These results infer a lower risk among higher carbohydrate intake as compare to lower carbohydrate intake.

**Table A:** Association between carbohydrate intake and chronotypes

	1^st^ Quintile	2^nd^ Quartile	3^rd^ Quartile	4^th^ Quartile
Model 1: Exp(B) P-Value	1	0	-0.08 < 0.001	-0.16 < 0.001
Model 2: Exp(B) P-Value	1	0	-0.08 < 0.001	-0.17 < 0.001
Model 3: Exp(B) P-Value CI	1	-0.05 < 0.001	-0.09 < 0.001	-0.16 < 0.001

Model 1: Carbohydrate intake andCVR Model 2: Adjustment with age, sex, andrace. Model 3: Adjustment with age, sex, race, smoking status, alcoholconsumption, sleeping duration, BMI, waist circumference, triglycerides, systolic anddiastolic bloodpressure.

**Appendix (B): reliability test between chronotype classified by wake-up time and sleep onset**

We used the Cohen’s Kappa test to analyze the reliability of classifying the chronotypes either by using the sleep onset time or the wake-up time. The results of our analysis show a significant agreement towards the reliability (β= 0.2, P-value < 0.001), inferring that using the sleep offset or the wake-up time would not alter the chronotypes classification.

**Table B:** Agreement on the chronotypes classification by sleep onset time or wake-up time

Number ofcases 6,325	
Measurement of agreement kappa(β)	0.2
P-value	< 0.001
